# Management of a Complicated Crown Fracture in a 16-Year-Old Patient: A Case Report

**DOI:** 10.3390/reports8030132

**Published:** 2025-08-01

**Authors:** Ralitsa Bogovska-Gigova

**Affiliations:** Department of Pediatric Dentistry, Faculty of Dental Mediciene, Medical University of Sofia, 1431 Sofia, Bulgaria; r.bogovska@fdm.mu-sofia.bg

**Keywords:** dental injuries, crown fracture, dental trauma, fragment reattachment, endodontic treatment, pediatric dentistry

## Abstract

**Background and Clinical Significance**: Traumatic dental injuries, particularly complicated crown fractures of permanent incisors, are common in adolescents, with maxillary central incisors most frequently affected due to their prominent position. These injuries, often resulting from sports or accidents, require prompt management to prevent complications such as pulp necrosis or infection, which can compromise long-term prognosis. Fragment reattachment offers a conservative, esthetically favorable approach when the fractured segment is intact, with outcomes comparable to composite restorations. This case report underscores the importance of timely intervention and advanced restorative techniques in pediatric dentistry. **Case Presentation**: A 16-year-old male presented with a complicated crown fracture of the upper left central incisor sustained during a soccer game. The fracture extended subgingivally with pulp exposure. The patient preserved the fragment in saline. Treatment involved fragment reattachment using a dentin bonding agent and flowable composite resin, followed by single-visit root canal therapy due to delayed presentation (48 h). A glass fiber post was placed to reinforce the restoration due to significant coronal loss. Three years of follow-up visits (1, 3, 6, 12, 24, and 36 months) revealed no clinical or radiographic complications, with the tooth remaining asymptomatic and functional. **Conclusions**: This case underscores the effectiveness of fragment reattachment when combined with meticulous technique and long-term monitoring.

## 1. Introduction and Clinical Significance

Dental injuries are a common occurrence in childhood, with epidemiological studies indicating that up to 25% of school-aged children experience some form of dental trauma; the incidence is even higher in preschool years [1,2]. The most frequently affected teeth are the maxillary central incisors, both in the primary and permanent dentitions, reflecting their prominent position in the dental arch and vulnerability during falls or collisions [2,3,4]. These injuries commonly occur in home and school environments, with sports-related activities being a significant contributing factor, particularly in adolescents [5]. The clinical spectrum of dental injuries in children ranges from enamel fractures and luxation injuries to avulsions and soft tissue trauma. In the primary dentition, luxation injuries predominate, while crown fractures are more common in the permanent dentition [2,3]. Timely management is critical, as delays in treatment can adversely affect prognosis and increase the risk of complications such as pulp necrosis, infection, or developmental disturbances in the permanent teeth [6,7].

The American Academy of Pediatrics underscores the importance of anticipatory guidance, injury prevention, and prompt referral to dental professionals to optimize outcomes [8]. Effective management requires early intervention and long-term follow-up to mitigate both immediate and delayed sequelae, particularly those affecting the developing permanent dentition [7,9].

Crown fractures of permanent incisors are among the most common traumatic dental injuries in children and young adults and typically involve the maxillary central incisors. These fractures are classified as either uncomplicated (enamel and dentin involvement without pulp exposure) or complicated (with pulp exposure) [10,11]. Uncomplicated crown fractures generally have a favorable prognosis, especially with no associated luxation injury [11]. The primary treatment is restoration with composite resin or adhesive reattachment if the fragment is available and intact [12,13]. Composite restorations demonstrate higher survival rates compared to fragment reattachments, though both are considered reliable options [14].

The longevity of restorations for fractured teeth due to trauma is influenced by factors such as the presence of luxation, the extent of the fracture, and the restoration technique [12,13,15]. Root canal treatment is reserved for cases where pulp necrosis develops or when the tooth is mature and pulp preservation is not feasible [16]. Concomitant luxation injuries significantly increase the risk of pulp necrosis and negatively impact prognosis [12,16]. Close follow-up is essential, especially within the first two years post-injury, as most complications (such as pulp necrosis or restoration failure) occur during this period [16]. Overall, with appropriate management, the prognosis for crown-fractured permanent incisors is good, but outcomes are optimized by early intervention and regular monitoring [12,16].

The reattachment of a tooth crown (fragment reattachment) is a conservative restorative procedure indicated when a fractured tooth fragment is available, intact, and fits well into the remaining tooth structure [17]. The process begins with a thorough clinical and radiographic assessment to determine the extent of the fracture and rule out luxation or root involvement [17,18]. If the pulp is exposed (complicated fracture), endodontic therapy is typically required prior to reattachment; for uncomplicated fractures, direct reattachment may proceed if the pulp is vital and asymptomatic [19].

The fragment should be stored in a hydrating medium (e.g., saline) to preserve its properties. The fragment and the remaining tooth are cleaned, and the fit is verified. Isolation with a rubber dam is recommended to prevent microbial contamination [20]. The bonding surfaces are often prepared with minimal or no additional preparation, although techniques such as beveling, chamfering, or internal grooves may enhance retention and strength [19,21].

An adhesive system is applied to both the fragment and the tooth remnant. A flowable or micro hybrid composite resin is commonly used as the intermediate material, providing both optimal bond strength and esthetics [20,22,23]. The fragment is positioned and light-cured. In cases with significant loss of tooth structure or after endodontic treatment, a fiber-reinforced post may be placed to support the fragment [18,19]. Postoperative care includes occlusal adjustment and regular follow-up to monitor for complications such as pulp necrosis, detachment, or discoloration. Long-term studies indicate favorable outcomes with this approach, with survival rates comparable to direct composite restorations [24,25].

In Bulgaria, where dental trauma is prevalent due to sports and accidents, effective management strategies are critical to improving oral health outcomes [26,27]. This case report details the treatment of a 16-year-old patient with a complicated crown fracture of the upper central incisor. This article provides a clinical case of crown fracture with reattachment, endodontic treatment, and placement of a fiber post, and an overview of the epidemiology, classification, and management of dental injuries in children, with a specific focus on crown fractures of permanent incisors and the technique of fragment reattachment.

### Clinical Significance

This case and the broader study of dental trauma management hold significant clinical implications for pediatric dentistry. In this case, the successful management of a complicated crown fracture underscores the viability of fragment reattachment as a conservative, esthetically favorable approach when the fragment is intact and properly preserved. Using a fiber-reinforced post addressed the structural compromise caused by significant tooth loss and endodontic treatment, aligning with evidence that such reinforcement enhances restoration longevity. The long-term outcome (3 years) supports the literature indicating high survival rates for reattachment when performed with meticulous technique.

The case highlights the importance of timely intervention. Educational campaigns targeting schools and sports clubs could reduce the incidence of trauma through protective measures like mouthguards.

This case illustrates the technical demands of managing complicated fractures, including endodontic therapy and fiber-post placement, which require advanced training. This case’s success suggests that continuing education in advanced restorative techniques could enhance treatment quality and increase dentists’ willingness to manage pediatric trauma cases.

## 2. Case Presentation

A 16-year-old male patient presented to Dental Praxis Dr Anton Gigov Dental Studio (Sofia, residential complex Krasno Selo, Kjustendil street 51, 1612, Bulgaria) 48 h after sustaining maxillofacial trauma during a soccer game. The patient, treated by Dr. Ralitsa Bogovska-Gigova, reported moderate pain and thermal sensitivity in the upper left central incisor (tooth 21). Medical history was unremarkable, with no allergies or systemic conditions.

### 2.1. Clinical Diagnosis

**Figure 1:**
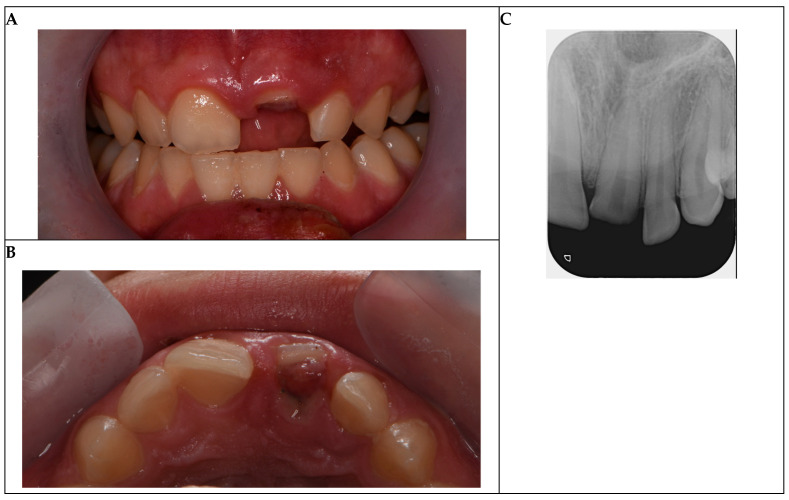
Initial situation. (**A**) Buccal view of the complicated crown fracture of tooth 21, showing subgingival extension and pulp exposure. (**B**) Occlusal view of the fractured tooth. (**C**) Pre-operative periapical radiograph confirming intact periodontal ligament space, mature root apex, and no periapical pathology.

Clinical examination revealed an Ellis Class III complicated crown fracture of tooth 21, characterized by an oblique fracture extending 2 mm subgingivally beyond the junctional epithelium, with visible pulp exposure (Figure 1A,B). The pulp exposure was approximately 1.5 mm in diameter, exhibiting prolonged bleeding (>2 min) and slight grayish discoloration, suggesting bacterial contamination and irreversible pulpitis due to the 48 h delay. Periodontal probing indicated a localized pocket depth of 4 mm at the fracture site, with healthy 2–3 mm depths elsewhere, confirming no generalized periodontal disease. Mobility testing showed grade 0 mobility (Miller classification), indicating intact periodontal support. Occlusion was evaluated using articulating paper, revealing no premature contacts or interferences in centric occlusion or lateral excursions. Magnified visual inspection with dental operation microscope (Semorr 3000E, Semorr Medical Tech Co., Suzhou, China) confirmed no additional cracks or fracture lines extending apically or to adjacent teeth (11 and 22).

Periapical radiographs, used as a diagnostic aid, confirmed complete root formation, a mature closed apex, and an intact periodontal ligament space with no periapical pathology or damage to adjacent teeth (Figure 1C). The oblique fracture line was visible subgingivally, with no evidence of vertical root fractures or alveolar bone involvement. The preserved crown fragment, stored in saline for one day before the appearance in the dental praxis, was intact and suitable for reattachment.

Given the prolonged pulp exposure, subgingival fracture extent, and signs of pulp compromise, vital pulp therapy was deemed unsuitable due to the high risk of bacterial contamination and limited pulp regeneration potential in a mature root. Single-visit root canal treatment (RCT) was indicated to prevent infection and ensure long-term prognosis.

Initial photographs and radiographs are presented in Figure 1.

### 2.2. Treatment Protocol

This treatment plan was designed to address pulp exposure, restore esthetics and function, and ensure the long-term stability of tooth 21 following a coronal fracture. A fragment reattachment was planned, followed by single-visit root canal treatment (RCT) with fiber-post reinforcement to optimize structural integrity and esthetic outcomes. The detailed protocol is outlined below.

### 2.3. Cleaning and Disinfection

**Figure 2:**
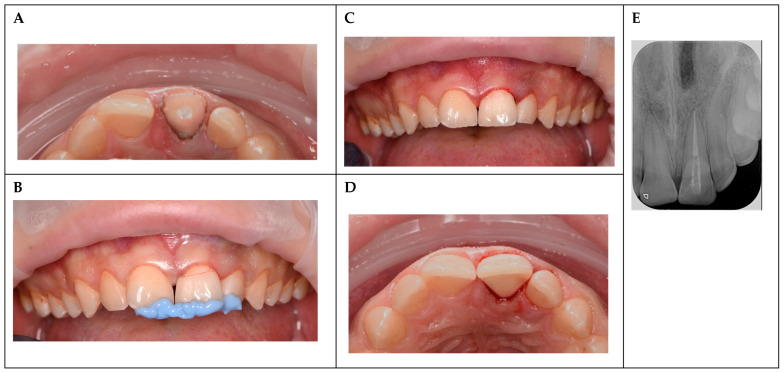
Treatment procedures. (**A**) Occlusal view after isolation. (**B**) Buccal view of the check-up fit of the placed fragment, with a liquid rubber dam guide in place. (**C**) Buccal view of the reattached fragment and restored tooth. (**D**) Occlusal view of the reattached fragment and restored tooth. (**E**) Periapical radiograph post-endodontic treatment and fiber-post placement, demonstrating hermetic root canal obturation and post stability.

Emergency treatment was initiated under local anesthesia (2% lidocaine with 1:100,000 epinephrine). The fracture site and exposed pulp were thoroughly irrigated with sterile saline followed by 2.5% sodium hypochlorite (NaOCl) to control the bleeding, disinfect the area, and remove debris, thereby minimizing the risk of infection. The subgingival fracture margin was exposed using electrosurgery to ensure adequate isolation (Figure 2A). Hemostasis was achieved using sterile cotton pellets. The fractured fragment was cleaned with 2% chlorhexidine gluconate and stored in isotonic saline to maintain hydration and prevent desiccation until reattachment.

### 2.4. Fragment Reattachment

Prior to reattachment, gingival tissues were gently retracted using retraction cords to ensure a clean and accessible operative field, minimizing contamination and enhancing visibility. The dentin surfaces of both the fragment and the tooth were prepared, and a dentin bonding agent (Prime & Bond NT, Nano-technology Dental Adhesive, Dentsply Sirona, Charlotte, NC, USA) was applied to ensure optimal adhesion. A flowable composite resin (Filtek™ Supreme XTE Flowable, 3M ESPE) was then used to secure the fragment, achieving precise adaptation and seamless esthetic integration (Figure 2D). Occlusion was rechecked post-reattachment to ensure no high spots.

### 2.5. Endodontic Treatment

Due to a 48 h delay between the trauma and treatment resulting in potential pulp contamination, a single-visit RCT was performed. The root canal was accessed and the pulp extirpated. Working length was determined using an electronic apex locator and confirmed radiographically. The canal was instrumented using rotary nickel–titanium files to an apical size of ISO 3630-1 40 (ProTaper Next, Dentsply Sirona), irrigated with 2.5% sodium hypochlorite and 17% EDTA, and dried with paper points. Obturation was performed using gutta-percha and an epoxy-resin-based sealer (AH Plus, Dentsply Sirona) to ensure a hermetic seal (Figure 2E). In this case, root canal treatment was performed without rubber dam isolation due to the subgingival extent of the fracture, which complicated clamp placement and achieving a stable seal. The fracture margin extended below the gingival level, and despite efforts to expose the margin using electrosurgery and retraction cords, the anatomical constraints and risk of gingival trauma precluded effective rubber dam application. To mitigate the risk of contamination, the operative field was meticulously managed through thorough irrigation with 2.5% sodium hypochlorite and 2% chlorhexidine gluconate, supplemented by sterile saline, to disinfect the area and remove debris. Retraction cords were used to maintain a dry and accessible field, and strict aseptic protocols were followed, including the use of sterile instruments and materials.

### 2.6. Fiber-Post Placement

A prefabricated esthetic glass fiber post (RelyX™ Fiber Post 3D Glass Fiber Post, 3M™ ESPE, diameter 1.1 mm) was selected to reinforce the coronal structure compromised by significant tooth loss. The post space was prepared within the root canal to a depth determined by the canal morphology and confirmed radiographically. The canal was etched with 37% orthophosphoric acid (3M™ Scotchbond™ Universal Adhesive, 3M ESPE) for 15 s, thoroughly rinsed with water, and dried with absorbent paper points (Meta Biomed, Cheongju, Republic of Korea). Excess water was removed to ensure a moist dentin surface. The adhesive (Prime & Bond NT, Dentsply) was applied to both the etched canal and the fiber post, air-thinned to remove excess, and light-cured for 10 s using a high-intensity LED curing light (Bluephase G4, Ivoclar Vivadent, Schaan, Liechtenstein). The fiber post was cemented using dual-cure resin cement (Multilink, Ivoclar Vivadent, Schaan, Liechtenstein) and light-cured for 40 s to ensure complete polymerization and robust structural support (Figure 2E). The subgingival extent of the fracture complicated stable clamp placement for rubber dam isolation, and the potential risk of fracturing the newly reattached fragment during clamp application necessitated its omission.

The patient was prescribed ibuprofen (400 mg every 6 h as needed) for pain management and instructed to maintain a soft diet and oral hygiene, avoiding trauma to the restored tooth.

### 2.7. Follow-Up

A structured follow-up protocol was implemented with evaluations at 1, 3, 6, 12, 24, and 36 months post-treatment to ensure rigorous clinical control. Clinical assessments included evaluation of symptoms (pain, sensitivity), restoration integrity (marginal adaptation, absence of secondary caries), and periodontal health (probing depths ≤ 3 mm, no bleeding on probing). Radiographic examinations assessed periapical healing, root resorption, and the stability of the fiber post and reattached fragment.

**Figure 3:**
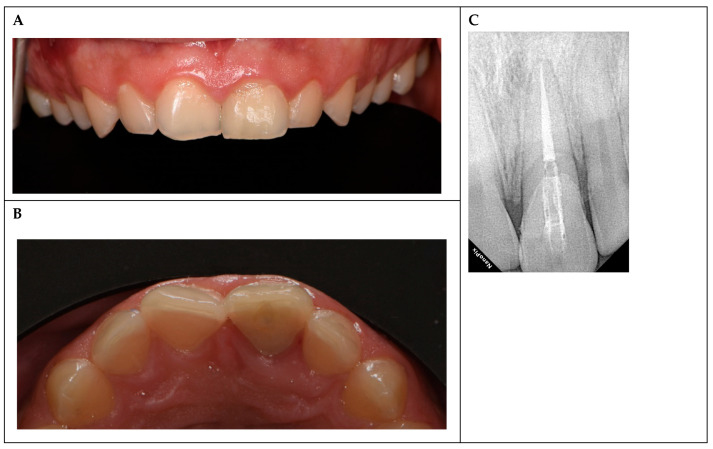
Three-year follow-up. (**A**) Buccal view showing intact restoration and esthetic outcome. (**B**) Occlusal view confirming restoration integrity and occlusal harmony. (**C**) Periapical radiograph at 36 months, indicating no periapical pathology and stable fiber post and restoration.

Throughout the follow-up period, both the endodontic and restorative treatments remained clinically acceptable. The tooth was asymptomatic, with no reported pain, sensitivity, or functional impairment (Figure 3A,B). Clinical examinations revealed intact restoration margins, no secondary caries, and stable periodontal parameters, including probing depths within normal limits (≤3 mm) and no bleeding on probing.

At 36 months, the tooth remained asymptomatic and functional, with intact restoration margins, stable periodontal health, and no radiographic evidence of periapical pathology or material degradation (Figure 3). These results confirm the clinical success of the treatment approach over the 3-year follow-up period. There were no signs of periapical pathology, such as radiolucency or bone loss, indicating successful endodontic treatment. The glass fiber post and composite restoration remained stable, with no evidence of debonding, fracture, or material degradation. The reattached fragment showed no signs of discoloration or structural failure, confirming the long-term esthetic and functional success of the treatment.

## 3. Discussion

The management of the complicated crown fracture in this 16-year-old patient underscores the efficacy of fragment reattachment as a conservative, esthetically favorable, and functionally reliable approach for treating dental trauma in adolescents. This case aligns with the established literature emphasizing timely intervention, meticulous technique, and long-term follow-up to achieve optimal outcomes in complicated crown fractures of permanent incisors [13,28]. The successful 3-year outcome, with no evidence of periapical pathology, restoration failure, or discoloration, supports the long-term viability of fragment reattachment when the fractured fragment is intact and properly preserved. This is consistent with studies reporting the survival rates of reattached fragments as being comparable to or exceeding those of composite resin restorations over similar timeframes [13,28,29]. For instance, Yilmaz et al. reported a survival rate of 84% for fragment reattachment in complicated crown fractures over 3–5 years, attributing success to meticulous bonding techniques and fiber-post reinforcement [30]. Similarly, de Castro et al. documented a case of successful fragment reattachment in a young patient, with stable outcomes at 7 years, reinforcing the applicability of this technique in adolescents [31].

The decision to perform single-visit RCT was driven by the 48 h delay in presentation, subgingival fracture extent with pulp exposure more than 1 mm, and clinical signs of pulp contamination (prolonged bleeding and discoloration), which increased the risk of irreversible pulpitis or necrosis [32]. According to the guidelines, vital pulp therapy is less predictable for mature teeth with prolonged pulp exposure (>24–48 h) due to bacterial infiltration [9,10]. In this case, the mature root apex and significant coronal loss further supported RCT, followed by fiber-post placement to enhance structural integrity [19]. These factors highlight the importance of tailoring treatment to the specific clinical presentation and injury characteristics.

Preservation of the fractured fragment in a hydrating medium (saline) was critical to maintaining its physical properties and bonding potential, as dehydration can compromise the fragment’s color and bond strength [18]. The use of minimal preparation, flowable composite resin, and fiber-post reinforcement optimized both esthetics and function, consistent with best practices for fragment reattachment [23]. However, the management could be critiqued for not exploring alternative restorative options, such as direct composite restoration, which may be more feasible in cases where fragment preservation is suboptimal or isolation is challenging. While fragment reattachment offers superior esthetics by preserving the natural tooth structure, composite restorations can provide comparable durability in subgingival fractures and may be less sensitive to technique [25]. The choice of fragment reattachment in this case was justified by the intact fragment and adequate isolation, but the literature suggests that operator experience and case selection are critical to success [13].

The long-term follow-up protocol was crucial for monitoring complications such as pulp necrosis, detachment, or discoloration, which are most likely to occur within the first two years after injury [33]. The absence of adverse outcomes at the 3-year mark reinforces the reliability of the chosen approach and the importance of regular monitoring to ensure the restoration’s stability and the health of the supporting structures [34].

The 3-year follow-up results indicate a favorable prognosis for the treated tooth, with no clinical or radiographic complications, including periapical pathology, restoration failure, or discoloration. These outcomes align with the findings of others [30,35], who report survival rates exceeding 84% for fragment reattachment in complicated crown fractures over 3–5 years when meticulous bonding techniques and fiber-post reinforcement are employed. The absence of periapical pathology, restoration failure, or discoloration supports the long-term reliability of the treatment, though continued monitoring is essential to detect potential late complications such as debonding or root resorption, particularly given the subgingival fracture extent [13].

Pulp necrosis and root canal infections following root fractures are uncommon in young patients with wide root canals, contributing to favorable prognoses with appropriate management [36]. Although this case involves a complicated crown fracture rather than a root fracture, the principle of favorable healing in adolescents, as seen in our 16-year-old patient with a mature root apex treated with fragment reattachment and RCT, supports the observed success. The subgingival extent of the fracture necessitates ongoing monitoring to detect potential late complications, such as debonding or root resorption, which are critical for long-term restoration longevity [24]. Sobczak-Zagalska et al. emphasize the importance of long-term follow-up in young patients to plan management strategies for potential complications, reinforcing the need for continued observation in this case [36].

The spectrum of traumatic dental injuries (TDIs) includes enamel–dentin fractures, crown–root fractures, root fractures, and various types of luxation (concussion, subluxation, lateral, extrusive, and intrusive luxation), as well as avulsion [37,38]. Fractures, particularly crown fractures without pulp exposure, are the most prevalent injury type, while luxation injuries are also common, especially in younger patients. Combination injuries (fracture plus luxation) occur in about one-third of cases and are associated with a higher risk of complications such as pulp necrosis [33]. Pulpal complications are a major concern following TDIs. Pulp necrosis is the most frequent sequela, particularly in teeth with completed root formation and in cases of severe luxation or avulsion. Other complications include pulp canal obliteration and various forms of root resorption (inflammatory, replacement/ankylosis). The risk and type of complication depend on the injury type, severity, and stage of root development at the time of trauma [39].

The management of entire crown fractures in permanent teeth, when the fractured crown fragment is available and root canal treatment is indicated (typically due to pulpal involvement), involves a multidisciplinary approach [38]. The protocol generally includes endodontic therapy, followed by adhesive reattachment of the fragment, often with the use of a fiber post to enhance retention and stress distribution [13]. This approach is minimally invasive and preserves natural tooth structure and esthetics [38]. Clinical outcomes for this strategy are generally favorable, with multiple case series and systematic reviews reporting good functional and esthetic results over follow-up periods ranging from several months to several years, provided that the fragment is intact and the clinical conditions (e.g., ability to achieve isolation, absence of extensive subgingival fracture) are suitable [39]. The use of a fiber post is common, especially in cases with significant loss of coronal structure, and is associated with improved retention and fracture resistance [40]. Adhesive systems (total-etch, self-etch, or self-cure) and resin-based materials are typically used for reattachment.

This case demonstrates the clinical success of fragment reattachment combined with endodontic treatment and fiber-post reinforcement in managing a complicated crown fracture. It emphasizes the importance of timely intervention, proper fragment preservation, and long-term follow-up to achieve favorable outcomes. By integrating evidence-based techniques and preventive strategies, dental professionals can enhance the management of dental trauma in children and adolescents, improving both functional and esthetic outcomes. Reattachment of the fractured crown fragment with root canal treatment is a conservative and esthetically superior option with generally good outcomes but requires careful case selection, meticulous technique, and regular follow-up to monitor for restorative or biological complications.

## 4. Conclusions

The described case illustrates the effective management of a complicated crown fracture in a 16-year-old patient through a combination of root canal treatment, fragment reattachment, and fiber-post placement. This evidence-based approach, which is well-supported by the literature for mature teeth with compromised pulp vitality, prioritizes conservative restoration to preserve both function and aesthetics. Prompt intervention and tailored treatment ensured optimal outcomes, underscoring the value of standardized protocols and consistent long-term monitoring to sustain tooth integrity and prevent complications in traumatic dental injuries.

## Data Availability

The original contributions presented in this study are included in the article. Further inquiries can be directed to the corresponding author.

## Abbreviations

The following abbreviations are used in this manuscript:

RCT	Root canal treatment
TDIs	Traumatic dental injuries
